# Foreign body appendicitis: A case report and literature review

**DOI:** 10.1097/MD.0000000000043214

**Published:** 2025-07-11

**Authors:** Mei-Chuan Liang, Chung-Hsin Chang, Sheng-Yang Huang, Chung-Wang Ko

**Affiliations:** a Department of Internal Medicine, Dalin Tzu Chi Hospital, Chiayi, Taiwan; b Buddhist Tzu Chi General Hospital Dalin Branch, Dalin Tzu Chi Hospital, Chiayi, Taiwan; c Department of Internal Medicine, Taichung Veterans General Hospital, Taichung, Taiwan; d Department of Internal Medicine, Division of Gastroenterology and Hepatology, Taichung Veterans General Hospital, Taichung, Taiwan; e Department of Pediatric Surgery, Taichung Veterans General Hospital, Taichung, Taiwan.

**Keywords:** foreign body appendicitis, screw appendicitis

## Abstract

**Rationale:**

Foreign body ingestion is more common in children than adults and occurs most frequently between the ages of 6 months and 6 years. While most foreign bodies pass spontaneously, retention in the appendix is rare and can lead to serious complications such as appendicitis or perforation.

**Patient concerns:**

We report the case of a 2-year 10-month-old boy referred to our pediatric gastroenterology division due to a witnessed ingestion of a screw, which remained in the gastrointestinal tract for 2 months.

**Diagnoses:**

Abdominal computed tomography revealed a screw-shaped metallic foreign body located near the ileocecal valve, with mild dilatation of the small bowel.

**Interventions:**

Given the prolonged presence of the foreign body in the right lower abdomen, a laparoscopic appendectomy was performed. Pathology confirmed early acute appendicitis.

**Outcomes:**

The patient recovered uneventfully and was discharged on postoperative day 5 without complications.

**Lessons:**

Although rare, appendicitis can result from foreign body ingestion. Serial abdominal X-rays or abdominal 3-dimensional computed tomography scans can assist in diagnosis. Management options include endoscopic retrieval or surgical removal, such as laparoscopic appendectomy.

## 1. Introduction

Foreign body ingestion occurs more often in children than in adults with most cases occurring in children aged 6 months to 6 years old.^[1]^ Most foreign bodies can be spontaneously passed through the gastrointestinal tract within 1 week and about 10% to 20% are removed by endoscopy. However, 1% of cases may require surgical intervention because of complications such as abscesses, peritonitis and perforation.^[2]^ Among the various types of foreign body ingestion, appendiceal foreign bodies are rare and may cause complication such as abscess, appendicitis and perforation. Patients may be asymptomatic for hours or years prior to the development of complications. We present a rare case of foreign body appendicitis in a 2-year 10-month-old boy who swallowed a screw and was managed by laparoscopic appendectomy.

## 2. Case report

**Figure 1.**
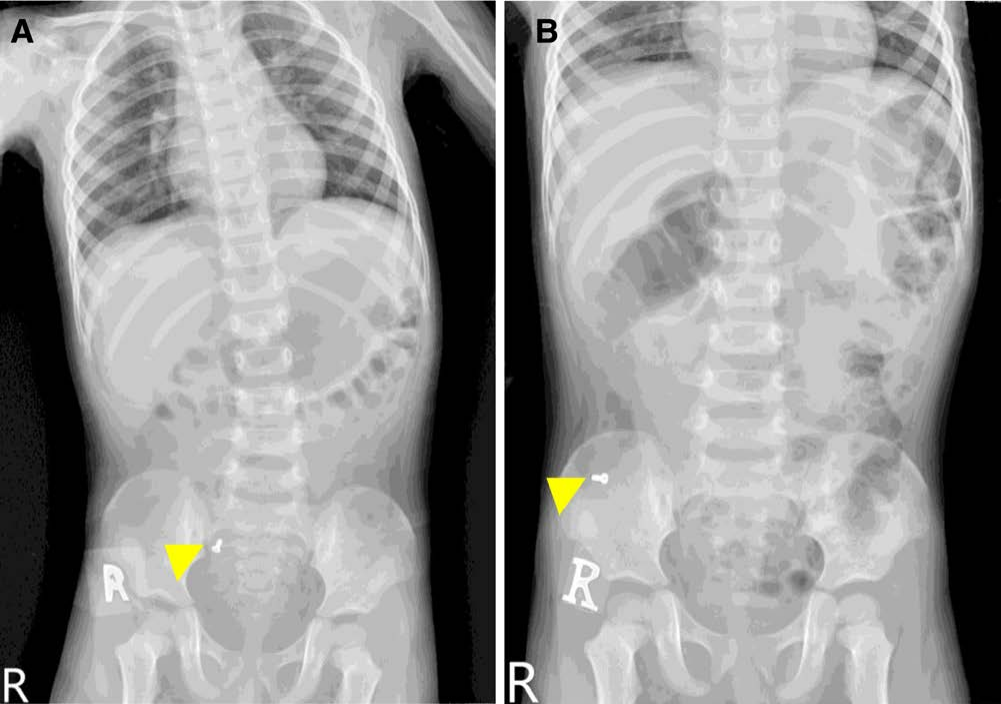
Foreign body screw (arrowhead) in right lower abdomen on (A) abdominal radiography and (B) serial abdominal radiography approximately 1 mo later.

**Figure 2.**
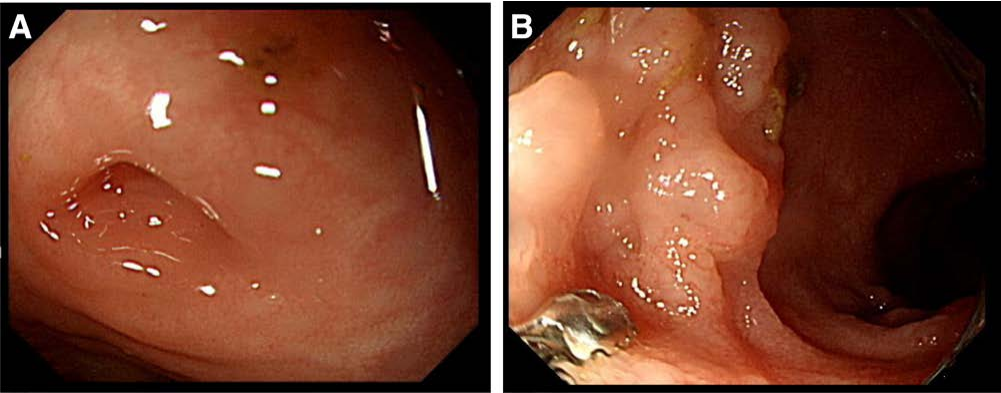
(A) No foreign body in appendix orifice. (B) Forceps approach in Peyer’s patches of terminal ileum.

**Figure 3.**
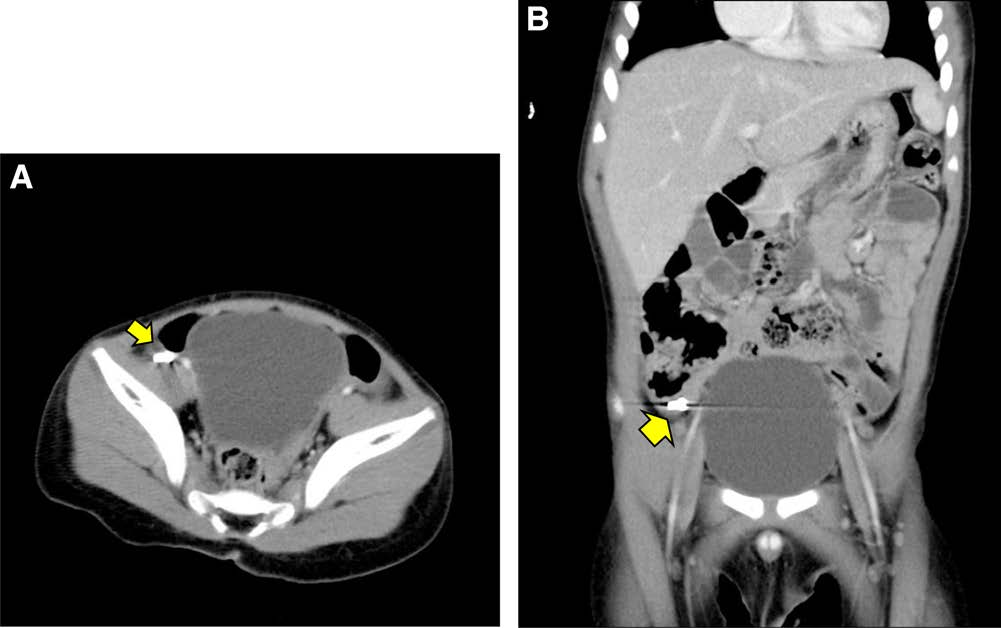
Screw shape metallic foreign body (arrow) over ileo-cecal valve with mild dilatation of small bowel under CT axial view (A) and coronal view (B).

A 2-year-10-month-old boy was referred to our pediatric gastroenterology division because of witnessed mis-swallowing of a screw with retention for 2 months. On admission, the boy was in a generally fair condition and denied symptoms including nausea, vomiting, abdominal pain, rebound tenderness or agitation. Physical examination revealed normoactive bowel sounds without abdominal tenderness or palpable mass lesion. Blood tests revealed normal hemogram, liver function and renal function. Serial abdominal radiography performed at a local hospital revealed that the screw was located in the right lower quadrant of the abdomen and measured 6mm in size (Fig. 1A and B). Enteroscopy was performed on April 10, 2023, in an attempt to remove the screw, but it could not be found from the terminal ileum to the cecum, despite thorough endoscopic ultrasound examination and the use of the biopsy forceps approach (Fig. 2A and B). Abdominal CT revealed a screw-shaped metallic foreign body over the ileocecal valve with mild dilatation of the small bowel (Fig. 3A and B).

**Figure 4.**
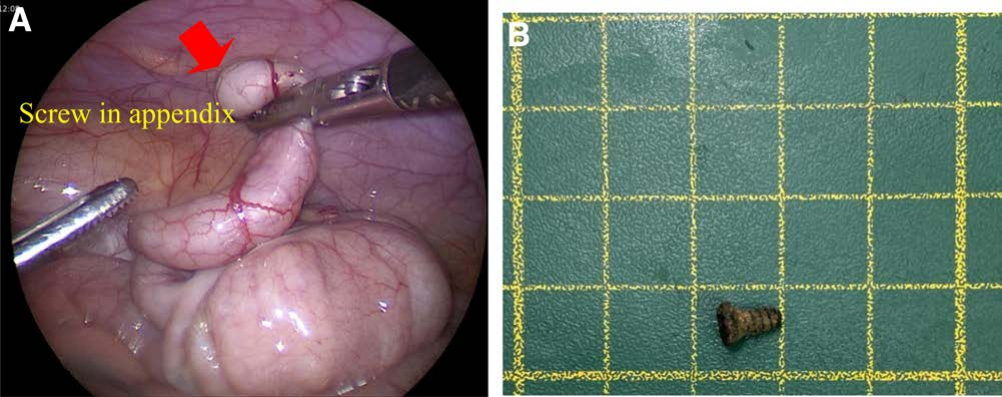
Inflamed appendix with the screw in lumen of appendix (A) and foreign body screw (B).

Exploratory laparoscopic surgery was performed to address concerns regarding the long-term risk of peritonitis caused by the screw. The position of the foreign body was checked using a C-arm with the assistance of laparoscopic instruments during the surgery. Laparoscopic appendectomy was performed after screw localization. The appendix was mildly inflamed with a screw in its lumen. The screw was embedded deep inside the appendix (Fig. 4A and B). Histopathological examination of the appendix revealed the features of early acute appendicitis. The patient was discharged on the 5th postoperative day. Thereafter, the patient was asymptomatic and was followed up in the outpatient department.

## 3. Discussion

We conducted a literature search and identified 11 case reports of foreign body appendicitis published between 2007 and 2022 (Table 1). Their ages varied from 11-months-old to 65-years-old. One patient had psychiatric disease. Most patients (N = 9) had typical symptoms and signs compatible with appendicitis; however 1 patient was diagnosed with an incidental finding and another patient had a globus sensation in the throat due to accidental swallowing of a drill bit.^[8,13]^ Foreign body appendicitis can occur within 1 week of the symptom onset. Two patients had leukocytosis however, most of them had normal blood test results.^[5,6]^ Abdominal CT scan was performed in most of the patients (N = 7), following the abdominal X-ray. Only 2 patients received colonoscopic retrieval using a magnetic device and Roth Net, and most patients underwent laparoscopic appendectomy.^[12,13]^

**Table 1:** Summary of case reports of foreign body appendicitis from 2007 to 2022.

Patient	Presentation	Physical examination	Laboratory examination	Diagnostic evaluation	Treatment
11-m-old-male baby^[3]^	2 d history of irritability, refusal to feed, fever and diarrhea	Tenderness and guarding in the right iliac fossa	Unknown	Abdominal X-ray: metallic screw in the RIF	Appendectomy
32-yr-old woman with tongue piercing ingestion^[4]^	RLQ abdominal pain	Tenderness in the RLQ area	Unremarkable	Abdominal X-ray:metallic foreign bodies overlying the cecum, CT: a metallic structure within the lumen of the cecum and another within the appendix or terminal ileum	Laparoscopic appendectomy
45-yr-old man with foreign body appendicitis^[5]^	Abdominal pain for 3 d	RLQ tenderness at right iliac fossa, muscle guarding	Leukocytosis	CT: acute appendicitis with obstruction of the appendiceal lumen by a foreign body measuring 1.7 cm in size distally	Laparoscopic appendectomy (Gangrenous AA)
20-yr-old man with needle ingestion^[6]^	Periumbilical pain and loss of appetite for 1 day	Tenderness in RLQ and periumbilical region	WBC: 14,000 × 103/μL	Abdominal X-ray: metallic foreign body in the right lower quadrant, CT: a needle in the appendix	Laparoscopic appendectomy
45-yr-old man with bizarre obstruction at appendix^[7]^	RLQ pain	Unremarkable	Unremarkable	Abdominal X-ray: radio-opaque formation in the right iliac fossa CT: a roundish-filling defect in right iliac fossa	Laparoscopic appendectomy
60-yr-old man with mis-swallowing of a drill bit at dental clinic^[8]^	Globus sensation in throat	Unknown	Unremarkable	Serial abdominal X-ray and contrast CT: intra-appendiceal foreign body with associated appendiceal distention and air retention, consistent with appendicitis	Laparoscopic appendectomy
2-yr-and-4-mo-old girl^[9]^	RLQ pain	RLQ tenderness with peritoneal irritation	Unremarkable	Abdominal X-ray: foreign body in RLQ abdomen	Transverse right lower quadrant incision appendectomy
4-yr-old boy with screw-induced appendicitis^[10]^	RLQ pain for 24 h, recurrent abdominal pain for 4 mo	Mild tenderness in right iliac fossa	Unremarkable	Plain roentgenogram: 1 metallic screw in right lower abdomen	Exploratory Laparotomy appendectomy
11-yr-old girl^[11]^	Abdominal pain for 3 d	Unknown	Unremarkable	CT: 2.7 cm radio opaque, linear object in the terminal ileum adjacent to the appendix	Diagnostic laparoscopy and appendectomy
56-yr-old Caucasian man^[12]^	Worsening abdominal pain and nausea for 2 d	Unremarkable	Unknown	Abdominal X-ray: foreign body in the right colon suggestive of a metal screw	Endoscopic removal with a Roth Net via colonoscopy
65-yr-old man with metallic screw^[13]^	Incidental finding	Unremarkable	Unremarkable	X-ray: radio opaque, sharp foreign body in the right lower quadrant, CT: screw in the appendiceal lumen	Endoscopic retrieval of appendiceal foreign body

Our patient was the third youngest patient with a diagnosis of foreign body appendicitis, and the rusted screw did not cause abdominal pain, even though the recalled time from mis-swallowing of the screw to diagnosis by X-ray was 3 months. Diagnostic enteroscopy was performed prior to the abdominal CT scan as the patient was not cooperative and the parents were concerned, which may have delayed the diagnosis. There is uncertainty regarding whether watchful waiting for an asymptomatic screw in the appendix would result in more severe complications.

1 Appendicitis caused by ingestion of the foreign bodies is rare. The incidence of foreign body appendicitis is only approximately 0.005% to 3% and numerous foreign bodies composed of various materials have been reported, including bird-shot pellets, screws, drill bits, needles, bone fragments, seeds, toothpicks, surgical staples and prosthetic material.^[3–5,10,11]^ However, screws are rarely present in the appendix. Foreign bodies may be immobile in the appendix for years without causing complications such as inflammatory response, peritonitis, abscess formation and perforation.^[6]^ Within the GI tract, the mechanism of foreign body appendicitis may be related to the dependent position of the cecum and its slower motility makes foreign bodies susceptible to dislodge in the appendix. Once in the appendix, it is difficult to expel foreign bodies back into the cecum because of insufficient peristalsis.^[4,7,8]^ Some ingested foreign bodies remain within the appendix without complications such as inflammation for many years. The occurrence of complications usually depends on the size and shape of the foreign bodies. Cases of appendicitis caused by blunt foreign bodies are due to obstruction of the appendiceal lumen for a long period of time and elongated or sharp bodies can cause perforation leading to peritonitis.^[6]^ Diagnosis of a foreign body in the gastrointestinal tract can be performed by serial abdominal X-ray follow-up or abdominal 3D computed tomography. If a foreign body is present in the cecum, endoscopic retrieval should first be considered. Laparoscopic foreign body removal is indicated to prevent complications such as perforation, peritonitis, and abscess formation if there is no progression of foreign body movement in the abdomen for more than 72 hours.^[9]^ Hence, laparoscopic management should be arranged for diagnostic and therapeutic purposes when retrieval endoscopy is not feasible owing to the risk of perforation or subsequent development of obstructive appendicitis.

## Acknowledgments

We would like to express our sincere appreciation to the Evidence-based Practice and Policymaking Committee of Taichung Veterans General Hospital for their valuable assistance in the preparation of this report.

## Author contributions

**Conceptualization:** Chung-Hsin Chang.

**Investigation:** Sheng-Yang Huang.

**Resources:** Sheng-Yang Huang.

**Supervision:** Chung-Wang Ko.

**Validation:** Chung-Wang Ko.

**Visualization:** Chung-Wang Ko.

**Writing – original draft:** Mei-Chuan Liang.

**Writing – review & editing:** Mei-Chuan Liang, Chung-Hsin Chang.
